# Effectiveness, cost-utility, and benefits of a multicomponent therapy to improve the quality of life of patients with fibromyalgia in primary care: A mixed methods study protocol

**DOI:** 10.1097/MD.0000000000017289

**Published:** 2019-10-11

**Authors:** Rosa Caballol Angelats, Alessandra Queiroga Gonçalves, Carina Aguilar Martín, Maria Cinta Sancho Sol, Gemma González Serra, Marc Casajuana, Noèlia Carrasco-Querol, José Fernández-Sáez, Maria Rosa Dalmau Llorca, Rosa Abellana, Anna Berenguera

**Affiliations:** a Equip d’Atenció Primària Tortosa Est, Institut Català de la Salut, Tortosa; b Unitat d’Expertesa en Sindromes de Sensibilització Central Terres de l’Ebre, Institut Català de la Salut, Tortosa, Tarragona; c Unitat de Suport a la Recerca Terres de l’Ebre, Fundació Institut Universitari per a la recerca a l’Atenció Primària de Salut Jordi Gol i Gurina (IDIAPJGol); d Unitat Docent de Medicina de Família i Comunitària Tortosa-Terres de L‘Ebre, Institut Català de la Salut; e Unitat d’Avaluació, Direcció d’Atenció Primària Terres de l’Ebre, Institut Català de la Salut; f Fundació Pere Mata Terres de l’Ebre; g Servei de Rehabilitació i Medicina Física, Hospital de Tortosa Verge de la Cinta, Institut Català de la Salut, Tortosa; h Fundació Institut Universitari per a la recerca a l’Atenció Primària de Salut Jordi Gol i Gurina (IDIAPJGol), Barcelona; i Universitat Autònoma de Barcelona, Bellaterra (Cerdanyola del Vallès; j Departament de Fonaments Clínics, Facultat de Medicina, Universitat de Barcelona, Barcelona, Spain.

**Keywords:** cost utility analysis, fibromyalgia, implementation science, pragmatic clinical trials, primary health care, qualitative research, quality of life

## Abstract

**Introduction:**

Fibromyalgia (FM) is a chronic condition characterized by chronic pain, fatigue and loss of function which significantly impairs quality of life. Although treatment of FM remains disputed, some studies point at the efficacy of interdisciplinary therapy. This study aims to analyze the effectiveness, cost-utility and benefits of a multicomponent therapy on quality of life (main variable), functional impact, mood and pain in people suffering from FM that attend primary care centers (PCCs) of the Catalan Institute of Health (ICS).

**Methods and analysis:**

A 2-phase, mixed methods study has been designed following Medical Research Council guidance. Phase 1: Pragmatic randomized clinical trial with patients diagnosed with FM that attend one of the 11 PCCs of the ICS *Gerència Territorial Terres de l’Ebre*. We estimate a total sample of 336 patients. The control group will receive usual clinical care, while the multicomponent therapy group (MT group) will receive usual clinical care plus group therapy (consisting of health education, exercise and cognitive-behavioural therapy) during 12 weeks in 2-hourly weekly sessions. Analysis: the standardized mean response and the standardized effect size will be assessed at 3, 9, and 15 months after the beginning of the study using multiple linear regression models. Utility measurements will be used for the economic analysis. Phase 2: Qualitative socio constructivist study to evaluate the intervention according to the results obtained and the opinions and experiences of participants (patients and professionals). We will use theoretical sampling, with 2 discussion groups of participants in the multicomponent therapy and 2 discussion groups of professionals of different PCCs. A thematic content analysis will be carried out.

**Ethics and dissemination:**

This study protocol has been approved by the Clinical Research Ethics Committee of the *Fundació Institut Universitari per a la recerca a l’Atenció Primària de Salut Jordi Gol i Gurina* (code P18/068). Articles will be published in international, peer-reviewed scientific journals.

**Trial registration:**

Clinical-Trials.gov: NCT04049006.

## 1 Introduction

Fibromyalgia (FM) is a disease characterized by chronic pain, fatigue, and loss of function, which physically limits the patient and consequently impacts mood, the social and working environments, generates sleep disturbances and generally impairs quality of life.^[1]^ FM is classified as a central sensitivity syndrome (CSS), and is frequently associated to other CSS such as Chronic Fatigue Syndrome and Multiple Chemical Sensitivity.^[2,3]^

The diagnosis of FM is based on clinical criteria, initially defined by the American College of Rheumatology.^[4,5]^ New amendments added in 2016 improve the detection of the disease in the primary care (PC) setting, although at the cost of lower specificity.^[6]^ Prevalence of FM in the general population ranges between 0.2% and 6.6%, and in women from 2.4% to 6.8%.^[7]^ In Spain, prevalence is estimated at 2.45% (D. Seoane-Mato, personal communication, July 2019), with a female/male ratio of 21:1, and is usually diagnosed in middle-aged people.^[1,8]^

The cost of FM for the health system is high.^[9]^ Total annual costs per patient in industrialized countries are estimated between 7256 and 7900 euros, and indirect costs (for instance, productivity loss) are considered higher than direct costs (such as medical visits and multiple prescriptions).^[10–12]^ In addition, when comparing with other conditions that cause chronic pain, FM is associated with higher rates of unemployment and a higher number of sick leave days.^[13]^

The best treatment for FM is currently disputed. Some studies have shown clinical improvement with non-pharmacological therapies, particularly with exercise^[14]^ and with cognitive-behavioral therapy (CBT).^[15,16]^ There is also some evidence on the effectiveness of multicomponent group therapy, which involves patient education, exercise and CBT^[17,18]^, with reported clinical improvement that lasts 3 to 12 months after the intervention.^[19,20]^ Interdisciplinary interventions conducted from hospitals^[21–23]^ and PC^[24–27]^ have obtained similar positive results. The most recent review (2017) of the European League Against Rheumatism (EULAR)^[28]^ on the management of FM endorsed the abovementioned therapies, and also included promising results on complementary therapies such as mindfulness, hydrotherapy and acupuncture. There is also evidence on the benefits of some drugs for the treatment of severe pain and insomnia (amitriptyline and cyclobenzaprine, duloxetine and pregabalin).^[28]^

Few studies on the economic evaluation of interventions for patients with FM have been published to date.^[29–33]^ With respect to cost-utility, psychology-based therapies showed better results in most studies.^[30–33]^ A cost-utility study carried out in Spain that compared a psychoeducational intervention with usual clinical care found that on average, the incremental gain in quality-adjusted life years (QALYs) per person was 0.12 (0.06 to 0.19).^[30]^

Complex interventions, as well as interventions based on Health Coaching, aim to promote healthy habits. The design of complex, multimodal, and interdisciplinary interventions aims to achieve a better implementation of interventions adapted to the people and their environment, and greater sustainability. The Medical Research Council has established a methodology to develop this type of interventions, which comprise various phases that are implemented iteratively using both quantitative and qualitative methodologies. The combination of methodologies incorporates the input of patients, in order to better adapt the intervention to their needs, and takes into account the characteristics (social, resources, health etc.) of the environment where the intervention takes place.^[34–36]^

In 2016, 18 accredited units specialized in Central Sensitivity Syndromes (USCSS) were created throughout Catalonia,^[37]^ with the aim to provide consistent health care to all patients with fibromyalgia.^[38]^ The USCSS consist of interdisciplinary teams based both in primary care centers (PCCs) and in hospitals.

After reviewing the available evidence, we want to propose a therapy for patients with FM to be delivered within the primary health care system. The objective of this proposal is to analyze the effectiveness and cost-utility of a multicomponent therapy that consists of health education, exercise and cognitive-behavioral therapy, and to compare it with usual clinical care with regard to quality of life, functional impact, mood and pain in patients with FM that attend Primary Health Care Centers (PCCs) of the *Gerència Territorial Terres de L’Ebre* of the Catalan Institute of Health (ICS). Based on the data collected about the opinion and experience of patients and professionals, the following aspects of the implementation of the intervention will also be evaluated: acceptability, adaptability, adherence, feasibility and compatibility. This intervention is founded on the transtheoretical model of change, which can be applied to multiple behaviors and risk factors and is recommended in research with a multibehavioral or multiple risk approach. Furthermore, the intervention applies the conceptual framework of the “5As” (Assess, Advise, Agree, Assist, and Arrange-follow up) to standardize and facilitate the intervention.^[39,40]^

## 2 Methods and design

### 2.1 Setting and participants

Participants will be patients diagnosed with FM that attend 11 PCCs of the ICS *Gerència Territorial Terres de l’Ebre*.

#### 2.1.1 Inclusion criteria (all criteria must be met)

- Clinical diagnosis of FM (International Classification of Diseases-10 codes: M79.0, M79.7);
- Having a phone number;
- Accepting participation in the study.

#### 2.1.2 Exclusion criteria

- Active psychotic episode;
- Intellectual impairment;
- Severe depression and personality disorder;
- Auto/heteroaggressive behaviour;
- Self-reported use of psychoactive substances;
- Having previously participated in a multicomponent therapy group (MT group) in the *Terres de l’Ebre* (pilot study);
- Unable to attend the group sessions.

### 2.2 Training of FM specialists

Before starting the study, a nurse or a general practitioner of each PCC (hereafter called “FM specialists”) will receive training on aspects related to FM, exercise, cognitive-behavioral therapy and on the methodology of data collection for the study. To guarantee quality and consistency, annual refresher training will be provided.

**Figure 1:**
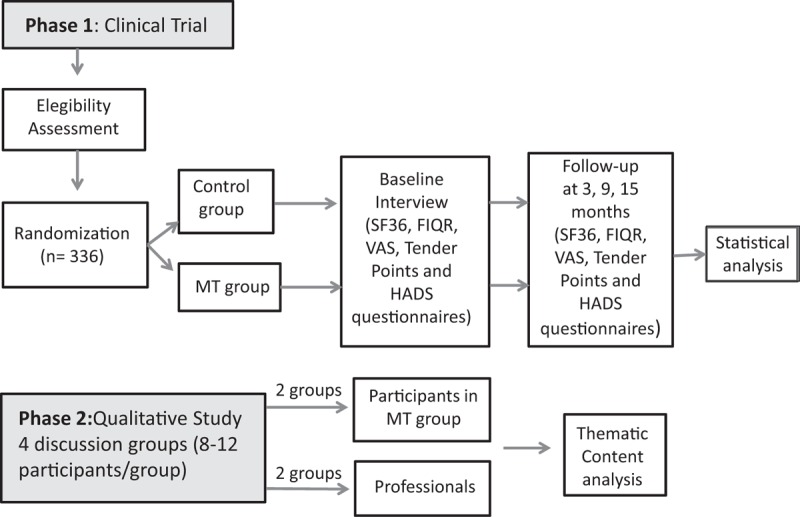
Flowdiagram of the study. FIQR = Revised Fibromyalgia Impact Questionnaire, HADS = Hospital Anxiety and Depression Scale, MT group = multicomponent therapy group, SF-36 = Short Form 36 health survey questionnaire, VAS = visual analog scale.

Following Medical Research Council guidelines^[35]^ on the evaluation of complex interventions, we will conduct a mixed methods study consisting of 2 phases. Figure 1 shows the study flowchart.

### 2.3 Phase 1: Study of the effectiveness and cost-utility of the intervention

#### 2.3.1 Study design and sample size

We will carry out a pragmatic randomized clinical trial (type: parallel group) with patients suffering from FM to compare the effect of a multicomponent therapy with usual clinical care in PC. The intervention will be structured in study units. Each study unit consists of a MT group and a control group, each with 10 to 12 participants. Assuming an alpha error of 0.05 and a beta error of 0.05 in a bilateral contrast, and an estimated dropout rate of 20%, a sample size of 130 participants in each group of the study will be required in order to detect a score difference equal or higher than 5 units in the Short Form 36 (SF-36) health survey questionnaire. A common standard deviation of 10 is assumed. Based on these calculations, we will create a total of 14 MT groups, with their respective control groups, between April and December 2019. The number of participants estimated is 336 patients.

#### 2.3.2 Randomization, recruitment, and data collection

For each study unit a randomization list will be generated according to Efron procedure,^[41]^ using the IBM SPSS Statistics v.23.0. package for Windows. An appointment will be arranged with patients who meet inclusion criteria for the first interview, where they will receive the information leaflet of the study and will sign the informed consent form. Next, patients will be allocated to a study group according to the randomization list. Additionally, the patients will be asked to respond to the study questionnaires (SF-36v2 health survey questionnaire^[42]^ (Optum, Inc. license number QM048943), Revised Fibromyalgia Impact Questionnaire (FIQR),^[43,44]^ Visual Analog Scale (VAS),^[45,46]^ tender points^[47]^ and Hospital Anxiety and Depression Scale (HADS)^[48,49]^) and will take part in the activities planned for each group (see below).

Control group: Patients in the control group will receive regular clinical care in their PCC,^[50]^ which consists on an individual visit with the FM specialist, who will conduct a standardized clinical history and will inform the patient on the disease using a custom designed leaflet.

**Figure 2:**
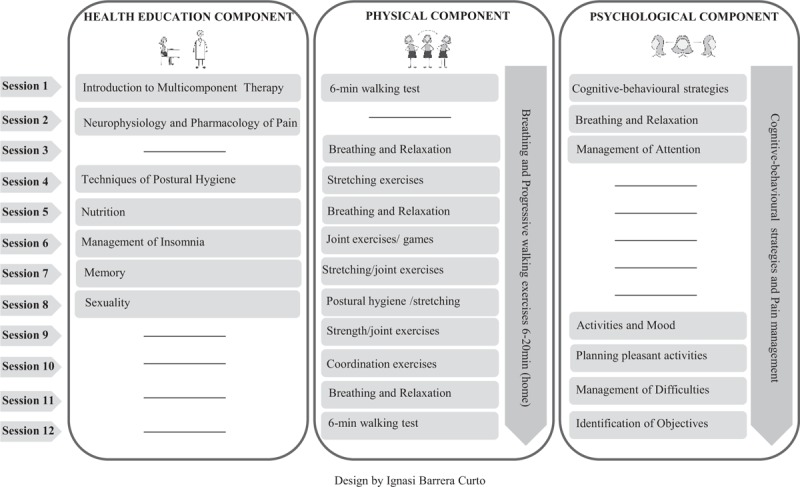
Content of the multicomponent therapy sessions.

MT group: in addition to usual PC clinical care, participants will receive MT consisting of health education, exercise and cognitive-behavioural therapy, during 12 weeks in 2-hourly weekly sessions (Fig. 2). Group therapy will be delivered by the FM specialist in each PCC, with the support of the physiotherapist and the psychologist of the USCSS.

Patients in the control and MT groups will receive an appointment to attend their PCC at 3, 9, and 15 months after the beginning of the study. During these appointments they will be asked to respond to the study questionnaires (SF-36, FIQR, VAS, tender points and HADS).

All data will be collected in a software application that has been custom designed for the treatment protocol of FM in the study sites. This application will be available in the *Terres de l’Ebre* ICS website and will be linked to the electronic medical records. The application aims to standardize all information related to patients with FM who visit the USCSS and will be used for research and for clinical care.

#### 2.3.3 Masking

Due to the study characteristics (pragmatic trial), it is not possible to mask the health professionals and participants. The data analyst will be blind to the patients’ allocation group.

#### 2.3.4 Outcomes

The main result variable of the study is quality of life (SF-36v2).^[42,51]^ Table 1 shows the study variables, the measurement instruments and the timing of data collection. All variables will be collected for both study groups. The cost-utility variables will be collected at 2 time points: 1 year before the beginning of the study and 1 year after the beginning of the study.

**Table 1:**
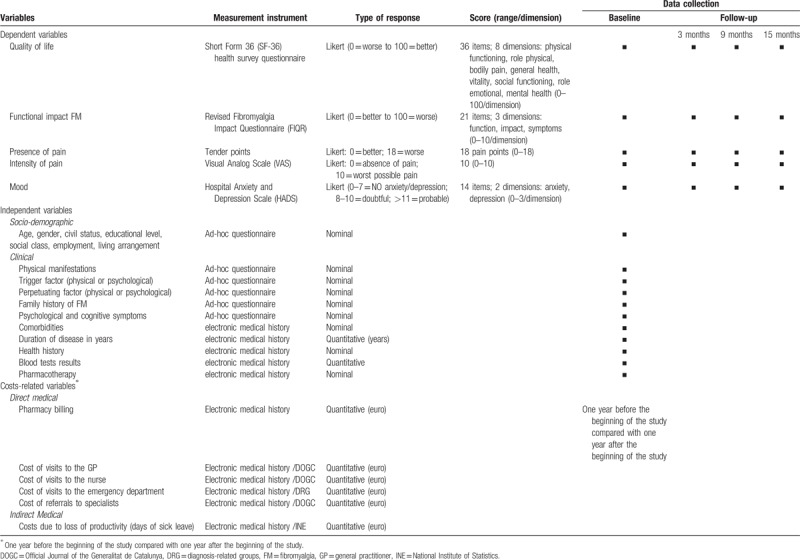
Study variables, measurement instruments and timing of data collection.

#### 2.3.5 Statistical analysis

Data analysis will take place at baseline and after 3, 9, and 15 months. Intention to treat analysis will be used. The quality of data collection will be periodically monitored to minimize biases due to incomplete data. The non-response bias will be evaluated at all points of follow up. Descriptive analyses of variables will be carried out using the media (standard deviation), median (interquartile range) or frequency (percentage), depending on the distribution of variables.

For comparisons between and within the 2 study groups (intervention and control) and the study variables, the following tests will be used: Student's *t* test for independent or paired data, McNemar, Chi-square test and analysis of variance or the corresponding non-parametric tests.

To evaluate the effectiveness of the intervention at each point of follow up, we will calculate the change in scores of the SF-36 and the other questionnaires in the intervention group minus the change in the control group, and the standardized effect size (SES). The SES will be calculated as the difference of the mean of the scores of both groups divided by the combined standard deviation. To detect differences within each study group, the difference of the means between baseline and each follow up point will be calculated, and also the effect size or standardized mean response (SMR), in accordance with Kazis’ method.^[52]^ The SMR will be calculated as the mean change divided by the standard deviation of change. To evaluate the SMR and the SES we will use Cohen rule, which classifies effect size as small (0.2 to 0.5), medium (0.5 to 0.8), and large (greater than 0.8).

Finally, multiple linear regression models will be used to explore the variables associated with the percentage change [(follow-up scores minus baseline scores)/baseline scores × 100] of the scores in the SF-36 and other questionnaires. The multivariate models will include variables that might be clinically linked, and the variables statistically significant in the previous bivariate analyses. Models will be also adjusted by confounding variables. Interactions and collinearity will be evaluated.^[53]^ Statistical significance for all study tests will be set at α ≤ 0.05 and 95% CI. The statistical package IBM SPSS Statistics v.23.0.for Windows will be used.

#### 2.3.6 Economic evaluation

The perspective of the National Health System will be used for the analysis of the economic evaluation. The use of resources and their respective costs for both groups will be described and compared. Temporal horizon will be 12 months, with a discount rate of 3%. Costs will be calculated according to the most recent official prices published in the *Diari Oficial de la Generalitat de Catalunya* (DOGC)^[54]^ for the public sector, updated to 2019. The utilities will be calculated using the SF-36v2, to obtain the quality-adjusted life years (QALYs) as effectiveness measure. The incremental ratio of the cost-utility will be calculated dividing the difference in total mean costs in both groups by the differences in QALYs. To check robustness of results we will carry out a sensitivity analysis with various discount rates and different costs. Table 1 shows costs-related variables, measurement instruments and timing of data collection.

### 2.4 Phase 2: Study of the evaluation of the intervention using qualitative methodology

The evaluation of the intervention will be founded on the constructs and guidelines suggested by experts of implementation research.^[55,56]^ The objective of this Phase is to evaluate the intervention with the use of qualitative methodology, with the aim to detect improvable aspects according to the opinion and experiences of participants (users and professionals).

#### 2.4.1 Study design

Interpretive, descriptive qualitative research will be carried out.

#### 2.4.2 Participants, sampling, and recruitment

Patients who have participated in 95% of the MT group sessions, and PC professionals. We will use theoretical sampling to achieve maximum discursive variability. Informants of different gender, age, socioeconomic status, geographical area and progression of disease will be selected amongst participants. Regarding PC professionals, we will include informants of both genders, different age groups and disciplines (general practitioners, nurses, psychologists, physiotherapists, rheumatologists and physical medicine and rehabilitation doctors).

#### 2.4.3 Data collection

Two discussion groups of participants and 2 discussion groups of professionals from different PCCs will be organized. These 4 groups of 8 to 12 people will follow a topic list. When necessary, additional discussion groups will be conducted to achieve discourse saturation. The discussion groups will be audio and video recorded prior informed consent and will be literally and systematically transcribed. Identifying data of the informants in the transcripts will be anonymized.

#### 2.4.4 Data analysis

The text corpus will include the transcripts of the discussion groups and the field notes of the groups’ observers. After successive readings of the sole text corpus and the formulation of pre analytical intuitions, a thematic interpretive content analysis will be carried out with the support of the Atlas-Ti programme and thereafter triangulated amongst the various members of the research team.^[57]^ The meanings will be interpreted and an explanatory framework will be created with the contributions of each type of informant (patients and professionals participating in the study).

### 2.5 Ethics and dissemination

This study protocol (version 1, 04/04/2018) has been approved by the Clinical Research Ethics Committee of the *Fundació Institut Universitari per a la recerca a l’Atenció Primària de Salut Jordi Gol i Gurina* (IDIAPJGol), on 25/04/2018 (code P18/068), in agreement with the Declaration of Helsinki/Tokyo. All participants will receive oral and written information about the study and Informed Consent will be obtained. The research team undertakes to invite patients in the control group to participate in the next MT group that takes place in their PCC, because this intervention is devised as a long-term healthcare commitment. Confidentiality of study subjects will be guaranteed, in agreement with the Organic Law of Personal Data Protection (03/2018 December 5, LOPD) and with the Regulation (EU) 2016/679 (April 27) of the European Parliament and Council of Data Protection (RGPD) and the national regulations of application. Any alteration of the study protocol will be submitted to the ethical committee of the IDIAPJGol for approval and will be published in clinical trials.gov.

The results of the study will be published in scientific journals and will be presented in national and international meetings. The results will be communicated to the patients of our health region by means of a meeting and also through the local and national media, and will be also disseminated to the general population.

## 3 Discussion

This study aims to provide evidence on the effectiveness of a multicomponent therapy implemented in the public primary care setting with the objective to improve the quality of life of patients diagnosed with FM.

The proposal of the study originates from a healthcare demand which requires interdisciplinary collaboration between general practitioners, nurses, psychologists, physiotherapists, rheumatologists and physical medicine, and rehabilitation doctors. The project will take into account the available health resources in PC, the perceptions of patients and professionals, and will also evaluate cost-utility.

Since current evidence suggests that multicomponent group therapy is the most successful treatment for FM,^[58–60]^ the emerging challenge for the health system is to implement this approach effectively and sustainably in PC. We believe that PC constitutes the ideal setting for this type of interventions, since it provides accessibility and long-term care. However, data from the primary care setting and data comparing the cost-effectiveness of treatments provided in PC versus hospital-based treatments remain scarce.

If this intervention is effective, we anticipate a swift transfer of results in our setting and in other regions of Spain with similar primary care systems. A common strategic national health plan would facilitate further expansion of this program. Moreover, since other European nations have already implemented the healthcare model organized in USCSS, they could also benefit from our findings. A pilot study conducted in rehabilitation centers in Belgian hospitals showed an improvement regarding the impact of FM, pain in tender-points and other health parameters at the end of a multicomponent therapy lasting 12 weeks that combined exercise and CBT, with the exception of patients with moderate depression.^[18]^ In this study improvement started after 6 weeks of therapy, but no information is provided on the duration of the beneficial effects.

One limitation of our project is that the group leaders cannot be blinded to treatment allocation. However, the data analyst will be blinded to the participants’ allocation group. In order to minimize follow-up variability, the group leaders will receive instruction before the start of the study, and subsequent annual refresher training. In addition, the educational and therapeutic contents of the group sessions will be standardized. Finally, all study units will consist of the same 3 professionals: the FM specialist of the PCC, a physiotherapist and one psychologist.

This study will provide novel scientific evidence, since few randomized clinical trials in this area of knowledge include qualitative methodology to evaluate multicomponent interventions.^[58]^ The qualitative phase of the project will contribute data for the evaluation of the acceptability and feasibility of the intervention, in accordance with the opinion and experiences of participants (patients with FM and health professionals). It will also contribute information on barriers and facilitators that can be used to design an intervention more flexible, dynamic, and adapted to the needs of participants.

## Acknowledgments

The authors are grateful to the following Departments from the ICS for their contribution in the development and implementation of the study: *Gerència Territorial ICS Terres de l’Ebre, Direcció d’Atenció Primària de Terres de l’Ebre* and *Unitat de Sistemes d’ informació de la Gerència Territorial Terres de l’Ebre*.

The authors also thank the members of the *Grup de recerca en Síndromes de Sensibilització Central a les Terres de l’Ebre* (SensiTEbre group) for participate in the data collection of the study: Adrià Suazo Ciurana (*Unitat d’informàtica, Gerència Territorial ICS Terres de l’Ebre)*, Albert Gómez Sorribes (Equip d’Atenció Primària (EAP)l’Ametlla de Mar-el Perelló, ICS), Blanca Cuevas Baticón (EAP Tortosa Est, ICS), Carolina Lopez Guerrero (EAP Flix, ICS), Cesar Urbina Nieto (EAP l’Amettla de Mar-el Perelló, ICS), Elisabet Martí Solé (EAP l’Aldea-Camarles-l’Ampolla, ICS), Gemma Batlle Escolies (EAP Amposta, ICS), Immaculada Matamoros Callarisa (EAP Sant Carles-Alcanar, ICS), Joan Estivill Bargalló (EAP Móra la Nova-Móra d’Ebre, ICS), Jordi Baucells Lluís (*Unitat d’informàtica, Gerència Territorial ICS Terres de l’Ebre)*, Josep Ausensi Estellé (EAP Terra Alta, ICS), M. Àngels López Guerrero (EAP Móra la Nova-Móra d’Ebre, ICS), Maria Fibla Reverté (EAP Amposta, ICS), Montserrat Martí Cavallé (EAP Amposta, ICS), Noemí Espuny Vallés (EAP l’Ametlla de Mar-el Perelló, ICS), Núria Buera Pitarque (EAP l’Aldea-Camarles-l’Ampolla, ICS), Nuria Piñana Suazo (EAP Terra Alta, ICS), Pilar Pérez Acín (EAP Flix, ICS), Susana Chavarria Jordana (EAP l’Aldea-Camarles-l’Ampolla, ICS).

## Author contributions

**Conceptualization:** Rosa Caballol Angelats, Alessandra Queiroga Gonçalves, Carina Aguilar Martín, Anna Berenguera.

**Data curation:** Rosa Caballol Angelats, Maria Cinta Sancho Sol, Gemma González Serra, Noèlia Carrasco-Querol, José Fernández-Sáez, Maria Rosa Dalmau Llorca, Rosa Abellana.

**Formal analysis:** Marc Casajuana, José Fernández-Sáez, Rosa Abellana.

**Funding acquisition:** Rosa Caballol Angelats, Carina Aguilar Martín, Anna Berenguera.

**Investigation:** Rosa Caballol Angelats, Alessandra Queiroga Gonçalves, Maria Cinta Sancho Sol, Gemma González Serra, Marc Casajuana, Noèlia Carrasco-Querol.

**Methodology:** Alessandra Queiroga Gonçalves, Carina Aguilar Martín, Marc Casajuana, Rosa Abellana.

**Project administration:** Rosa Caballol Angelats, Carina Aguilar Martín, Anna Berenguera.

**Supervision:** Alessandra Queiroga Gonçalves, Noèlia Carrasco-Querol, Maria Rosa Dalmau Llorca, Rosa Abellana, Anna Berenguera.

**Visualization:** Maria Rosa Dalmau Llorca.

**Writing – original draft:** Rosa Caballol Angelats, Alessandra Queiroga Gonçalves, Carina Aguilar Martín, Maria Cinta Sancho Sol, Gemma González Serra, Marc Casajuana, Noèlia Carrasco-Querol, José Fernández-Sáez, Anna Berenguera.

**Writing – review & editing:** Rosa Caballol Angelats, Alessandra Queiroga Gonçalves, Carina Aguilar Martín, Maria Cinta Sancho Sol, Gemma González Serra, Marc Casajuana, Noèlia Carrasco-Querol, José Fernández-Sáez, Maria Rosa Dalmau Llorca, Rosa Abellana, Anna Berenguera.
