# Effectiveness, cost-utility, and benefits of a multicomponent therapy to improve the quality of life of patients with fibromyalgia in primary care: A mixed methods study protocol: Erratum

**DOI:** 10.1097/MD.0000000000018263

**Published:** 2019-11-27

In the article, “Effectiveness, cost-utility, and benefits of a multicomponent therapy to improve the quality of life of patients with fibromyalgia in primary care: A mixed methods study protocol”,^[1]^ which appears in Volume 98, Issue 41 of *Medicine,* the how to cite this article information appears incorrectly and should be:

Caballol Angelats R, Gonçalves AQ, Aguilar Martín C, Sancho Sol MC, González Serra G, Casajuana M, Carrasco-Querol N, Fernández-Sáez J, Dalmau Llorca MR, Abellana R, Berenguera A; SensiTEbre group. Effectiveness, cost-utility, and benefits of a multicomponent therapy to improve the quality of life of patients with fibromyalgia in primary care: A mixed methods study protocol. Medicine (Baltimore). 2019 Oct;98(41):e17289.
